# Supplementary material for: Higher number of steps is related to lower endogenous progesterone but not estradiol levels in women
Source: PLoS One. 2024 Apr 4;19(4):e0299580. doi: 10.1371/journal.pone.0299580 (PMC10994375; doi:10.1371/journal.pone.0299580)
Supplement: S1 Table — (PDF) [file pone.0299580.s001.pdf]

Supplementary Table 1

Higher number of steps is related to lower endogenous progesterone but not estradiol levels in women

Kinga Słojewska, Andrzej Galbarczyk, Magdalena Klimek, Anna Tubek-Krokosz, Karolina Krzych-Miłkowska, Joanna Szklarczyk, Magdalena Mijas, Monika Ścibor, Grazyna Jasienska

Table 1. The association between physical activity and salivary estradiol (E2) and progesterone (P) levels after controlling for age and body fat %.

		**Linear association** Average daily number of steps			**Two groups** more or less than 10,000 steps			**Three groups** tertiles of daily number of steps		
N = 85		Exp(b)	95%CI	p	Exp(b)	95%CI	p	Exp(b)	95%CI	p
**Estradiol (pg/ml)**										
Daily number of steps		-0.000008	-0,00004; 0.00002	0.61						
≥ 10,000 steps[a]					-0.02	-0.10; 0.06	0.62			
Tertiles [b]	1st							0.03	-0.08; 0.15	0.59
	2nd							0.02	-0.09; 0.13	0.69
Age (years)		0.006	-0.013; 0.025	0.55	0.004	-0.01; 0.02	0.66	0.01	-0.01; 0.02	0.58
Body fat (%)		0.006	-0.007; 0.018	0.40	0.01	-0.01; 0.02	0.38	0.01	-0.01; 0.02	0.39
**Progesterone (pg/ml)**										
Daily number of steps		**-0.00007**	**-0.00012; -0.00002**	**0.01**						
≥ 10,000 steps[a]					0.12	-0.01; 0.26	0.08			
Tertiles [b]	1st							**0.23**	**0.04; 0.43**	**0.02**
	2nd							-0.07	-0.25; 0.12	0.47
Age (years)		0.03	-0.01; 0.06	0.1	0.02	-0.01; 0.05	0.21	0.02	-0.01; 0.06	0.20
Body fat (%)		-0.02	-0.04; 0.003	0.09	-0.01	-0.04; 0.01	0.18	-0.02	-0.04; 0.005	0.12

Note: Boldface indicates statistical significance ($p<0.05$).

Abbreviation: Exp(b), exponentiated coefficient; CI, confidence interval.

[a] Reference level: Women who took less than 10,000 steps

[b] Reference level: 3rd tertile

.
