# Supplementary material for: Higher number of steps is related to lower endogenous progesterone but not estradiol levels in women
Source: PLoS One. 2024 Apr 4;19(4):e0299580. doi: 10.1371/journal.pone.0299580 (PMC10994375; doi:10.1371/journal.pone.0299580)
Supplement: S2 Table — (PDF) [file pone.0299580.s002.pdf]

Supplementary Table 2

Higher number of steps is related to lower endogenous progesterone but not estradiol levels in women

Kinga Słojewska, Andrzej Galbarczyk, Magdalena Klimek, Anna Tubek-Krokosz, Karolina Krzych-Miłkowska, Joanna Szklarczyk, Magdalena Mijas, Monika Ścibor, Grazyna Jasienska

Table 2. The association between physical activity and salivary estradiol (E2) and progesterone (P) levels after controlling for age and muscle mass

		**Linear association** Average daily number of steps			**Two groups** more or less than 10,000 steps			**Three groups** tertiles of daily number of steps		
N = 85		Exp(b)	95%CI	p	Exp(b)	95%CI	p	Exp(b)	95%CI	p
**Estradiol (pg/ml)**										
Daily number of steps		-0.00001	-0.00004; 0.00002	0.53						
≥ 10,000 steps[a]					-0.02	-0.10; 0.06	0.69			
Tertiles [b]	1st							0.04	-0.08; 0.16	0.49
	2nd							0.02	-0.09; 0.13	0.73
Age (years)		0.01	-0.01; 0.03	0.40	0.01	-0.01; 0.02	0.50	0.01	-0.01; 0.03	0.41
Muscle mass (kg)		-0.01	-0.03; 0.01	0.48	-0.01	-0.03; 0.01	0.56	-0.01	-0.03; 0.01	0.42
**Progesterone (pg/ml)**										
Daily number of steps		**-0.0001**	**-0.00012; -0.00001**	**0.02**						
≥ 10,000 steps[a]					0.12	-0.02; 0.26	0.09			
Tertiles [b]	1st							**0.23**	**0.03; 0.42**	**0.03**
3	2nd							-0.06	-0.25; 0.12	0.50
Age (years)		0.02	-0.01; 0.05	0.17	0.02	-0.01; 0.05	0.27	0.02	-0.01; 0.05	0.21
Muscle mass (kg)		-0.004	-0.04; 0.03	0.83	-0.004	-0.04; 0.03	0.81	-0.01	-0.04; 0.03	0.69

Note: Boldface indicates statistical significance ($p<0.05$).

Abbreviation: Exp(b), exponentiated coefficient; CI, confidence interval.

[a] Reference level: Women who took less than 10,000 steps

[b] Reference level: 3rd tertile
