# Supplementary material for: Higher number of steps is related to lower endogenous progesterone but not estradiol levels in women
Source: PLoS One. 2024 Apr 4;19(4):e0299580. doi: 10.1371/journal.pone.0299580 (PMC10994375; doi:10.1371/journal.pone.0299580)
Supplement: Attachment — Submitted filename: Response to Reviewers.docx [file pone.0299580.s004.docx]

Krakow, 8^th^ January 2024

Dear Editors,

We would like to thank the Reviewers and Editors for their insightful assessment of the manuscript. We have tried to adequately answer all reviewers’ comments. We believe that our work has improved significantly, and we hope our manuscript will be considered suitable for publication.

**Reviewer #1:**

*Although mentioned in the limitation, please clarify if you asked about other physical activities unrelated to steps. For example, the Fitbit Alta HR wristband accelerometers cannot track biking in the step count. If women performed other activities not monitored by the device, it could significantly change the results.*

Response: We asked participants about other activities but decided not to take them into account in this publication. Due to the fact that there are official recommendations on how many steps a day should be taken to maintain health, we have decided to include only the average number of steps taken per day in this publication. Information about other activities was self-reported by participants and thus not very reliable and difficult to quantify. In addition, the purpose of this study was to assess if number of steps per day correlates with levels of ovarian hormones. This approach allows to formulate conclusions that are useful for individual women and important in the context of public health in the area of breast cancer prevention.

*Table 1 should be in the results section*

Response: Thank you. Agreed. We moved Table 1 to the results section.

*Statistical analysis: “multiple regression models were used to test the effects of exercise (expressed as number of steps) on estradiol and progesterone levels.” I would consider changing it to “… (expressed as the average number of steps per day)”*

Response: Thank you for pointing out this shortcut. As suggested, we changed "expressed as number of steps" to "expressed as average number of steps per day".

*Table 2 – linear association between daily number of steps and estradiol level: I think the p-value should be 0.688 or 0.69 and not 0.60.*

Response: Thank you very much for spotting this mistake. We changed the p-value to 0.69.

*The reference to Feehan et al on page 19 should be changed to [49].*

Response: We are not entirely sure if our interpretation of the Reviewer’s suggestion is correct. We have found an incorrect style of the reference “Feehan et al., 2018” on page 12. We have now changed it to the Vancouver style. We would be grateful if the Reviewer could confirm if this was the suggested issue.

**Reviewer #2**

*Two subgroups of women were formed on the basis of the level of physical activity, however, the two subgroups are not described by age and the other studied biological parameters (height, weight, BMI) the description is given only for the whole sample (Table 1). Since the age-group is ranged between 20.4 and 35.4 years, this is important to describe. Sex hormone levels change by ageing, a decrease can be seen after 30 years of age in the level of estradiol and progesterone.* *The mean steps per day is 10319 in the whole sample, this value is rather high in this age-group, it should be important to see the mean value of steps per day also for the subgroups. The recommendation for the studied age group for steps per day is 7-10000 according to the literature (e.g. DOI:* [*https://doi.org/10.1016/S2468-2667(21)00302-9*](https://doi.org/10.1016/S2468-2667(21)00302-9)*). By considering the mean steps of the 2 subgroups could help in the understanding of the research results.*

Response: Thank you for pointing this out. We have supplemented Table 1 with descriptive characteristics of the subgroups. The current table included in the manuscript is presented below.

We agree, that the mean steps per day is rather high in this age-group. This is a result of the study's design. At the beginning of the observation, each woman received a fitness club pass which allowed for unlimited training sessions and was asked to perform no less than 180 minutes/week of moderate to vigorous physical activity of her choice. We have clarified this in the methods section.

**Table 1:** Baseline characteristics of study participants, their physical activity and sex hormone levels

|  | **All participants** | | | **Group** | |
| --- | --- | --- | --- | --- | --- |
|  | **Mean (SD)**  (N = 85) | **Min** | **Max** | **Less than 10,000 steps**  **mean (SD)**  (n = 39) | **More than 10,000 steps**  **mean (SD)**  (n = 46) |
| Age (years) | 27.1 (4.3) | 20.4 | 35.4 | 26.7 (4.0) | 27.5 (4.6) |
| Cycle length (days)^a^ | 29 (3.95) | 22 | 51 | 29.1 (4.67) | 28.9 (3.59) |
| Body height (cm) | 164.8 (5.5) | 146.7 | 183.8 | 166.0 (6.4) | 163.8 (4.3) |
| Body weight (kg) | 59.3 (8.34) | 44.7 | 90.3 | 60.4 (7.9) | 58.5 (9.2) |
| BMI (kg/m^2^)^b^ | 21.8 (2.85) | 16.3 | 32.1 | 21.9 (2.5) | 21.8 (3.1) |
| Average steps/day | 10319.3 (2703.6) | 3380.8 | 16011.0 | 7988.2 (1805.6) | 12295.8 (1479.2) |
| Estradiol (pg/ml) | 4,1 (1.7) | 2.1 | 11.6 | 4.0 (1.8) | 4.2 (1.7) |
| Progesterone (pg/ml) | 100.4 (62.6) | 15.2 | 303.9 | 113.0 (66.1) | 89.7 (58.1) |

^a^ during participation in the study

^b^ BMI, body mass index

*It is my impression that the cut-off value (10 000 steps/day) too high in the studied sample, or not only two but 3 subgroups should be formed (low, average, high level of physical activity) to analyse the relationship between the level of physical activity and sex hormone levels.*

Response: We decided to set a cut-off of 10,000 steps a day due to the widespread recommendations of this value as necessary to maintain health. However, we realize that there is evidence in the literature that health benefits are observed at rates lower than 10,000 steps per day. Therefore, we also divided the study group into 3 subgroups (tertiles: low = 8,209; medium = 10,450; high = 12,274) according to the average daily number of steps. These results are presented in Table 2.

*It is not clear why it is of high importance to ‘reduce’ the sex hormone levels in premenopausal women (this is emphasized by the Authors in the Abstract and in the main part of the manuscript, too). They are in the reproductive stage of their lives, decreased sex hormone levels can influence menstrual cycle, reproductive activity, bone health etc. It must be emphasized that the level of physical activity decreased very dramatically in the last decades, so increased level of physical activity means in this case that women have sufficient level of physical activity.*

Response: Indeed, this issue was not sufficiently explained in our original manuscript. We have added the following in the discussion:

It should be also emphasized that sex hormones, while related to the increased risk of breast cancer, are crucial for many aspects of healthy physiology [63,64]. Their beneficial role is well established for fertility, cardiovascular function, bone health and mental health. However, insufficient levels of physical activity may lead to changes in the body’s delicate hormonal balance contributing to altered hormone levels and adverse health outcomes. Thus, it is important for physically active women to discuss these issues with their physicians.

*Why only body weight measured by Tanita was analysed in the manuscript? This device estimate body mass components as well, why the relationship of sex hormone levels with body fat mass and muscle mass were not analysed? Increased body fatness is evidenced to be related with sex hormone levels in women.*

Response: Although the role of body mass index on sex hormone levels and the development of breast cancer in premenopausal women is not clear, body mass index has been shown to be associated with sex hormone binding [Ingram, D M et al. 1990; doi:10.1038/bjc.1990.57]. Thus, we decided to perform statistical analyzes using BMI because it is a parameter that anyone can easily calculate. We wanted our research to be understandable to people outside the scientific community and to be easily conveyed in conversation. This is important in the context of public health. Additionally, in response to the Reviewer’s comment we rerun our analyses replacing BMI with the percentage of body fat or muscle mass.
