# Supplementary material for: Higher number of steps is related to lower endogenous progesterone but not estradiol levels in women
Source: PLoS One. 2024 Apr 4;19(4):e0299580. doi: 10.1371/journal.pone.0299580 (PMC10994375; doi:10.1371/journal.pone.0299580)
Supplement: Attachment — Submitted filename: Response to Reviewers.docx [file pone.0299580.s005.docx]

Krakow, 8^th^ February 2024

Dear Editors,

We are glad that the additional information provided in the first response has made the article clearer and more comprehensive. At the same time, we thank you very much for the additional comments.

As suggested by the Reviewer #1, we have added results of independent samples t-tests to the Table 1. We have also stated in the Results section that: *There was no statistically significant differences between the groups of women who took less than 10,000 steps and more than 10,000 steps in mean age, cycle length, body height, body weight and BMI.*

We also made sure that we did not cite papers that have been retracted.

Yours sincerely,

Andrzej Galbarczyk

Max Planck Institute for Evolutionary Anthropology
